# Autophagy Roles in the Modulation of DNA Repair Pathways

**DOI:** 10.3390/ijms18112351

**Published:** 2017-11-07

**Authors:** Luciana R. Gomes, Carlos F. M. Menck, Giovana S. Leandro

**Affiliations:** Department of Microbiology, Institute of Biomedical Sciences, University of Sao Paulo, Sao Paulo 05508-900, SP, Brazil; luciana.gomes@usp.br (L.R.G.); giovanasl@usp.br (G.S.L.)

**Keywords:** autophagy, DNA repair, homologous recombination (HR), non-homologous end joining (NHEJ), base excision repair (BER), nucleotide excision repair (NER)

## Abstract

Autophagy and DNA repair are biological processes vital for cellular homeostasis maintenance and when dysfunctional, they lead to several human disorders including premature aging, neurodegenerative diseases, and cancer. The interchange between these pathways is complex and it may occur in both directions. Autophagy is activated in response to several DNA lesions types and it can regulate different mechanisms and molecules involved in DNA damage response (DDR), such as cell cycle checkpoints, cell death, and DNA repair. Thus, autophagy may modulate DNA repair pathways, the main focus of this review. In addition to the already well-documented autophagy positive effects on homologous recombination (HR), autophagy has also been implicated with other DNA repair mechanisms, such as base excision repair (BER), nucleotide excision repair (NER), and mismatch repair (MMR). Given the relevance of these cellular processes, the clinical applications of drugs targeting this autophagy-DNA repair interface emerge as potential therapeutic strategies for many diseases, especially cancer.

## 1. Introduction

Autophagy and DNA repair are biological processes essential to enabling a normal cell life. Consequently, changes in both mechanisms have been increasingly associated with a range of human disorders, making them important therapeutic targets [1]. The relevance of autophagy and DNA repair is already well recognized, and both mechanisms were recently the subjects of Nobel prize-winning works (in the areas of Physiology or Medicine in 2016 and Chemistry in 2015, respectively). Although they have long been regarded as independent processes, more and more evidence indicates that autophagy and DNA repair are, in fact, closely related [2,3,4,5,6,7].

Many reports have supported that, upon DNA damage, autophagy is stimulated and then modulates several events and molecules from the extensive DNA damage response (DDR) cascade [1,8], such as cell cycle arrest, cellular death rates and also the activity of the DNA repair machinery. The latter is the main focus of this review. Thus, below we discuss in detail how each of the DNA repair pathways are regulated by autophagy.

### 1.1. Autophagy and p53: How the Guardians of Proteome and Genome Relate to Each Other

A close and strong relationship between autophagy and p53 has been already established, and several of the autophagy effects in DDR are mediated by this transcription factor [9,10]. The tumor suppressor p53 exerts a dual role on autophagy modulation, depending on its location in the cell: the nucleus or cytoplasm [11]. When accumulated in the nucleus, p53 stimulates the autophagic process through direct transcriptional induction of the damage-regulated autophagy modulator (DRAM) [12]. In turn, cytoplasmic p53 is required for autophagy inhibition [13]. Besides being dependent on p53 subcellular localization, the bidirectional control of autophagy by p53 is also determined by the nature of the stress signal which triggers the cellular response [14]. Moreover, the p53 status can determine the role of autophagy in tumor development [15]. Thus, autophagy inhibition in tumors positive for p53 blocks their progression to high-grade stages. On the other hand, autophagy down modulation in a p53-null environment no longer prevents tumor progression, but actually accelerates this process [15].

Although it is a degradative process, there are no reports concerning the autophagy-mediated degradation of p53, since the wild-type p53 is a well-characterized target for ubiquitin proteasome system (UPS) degradation. However, mutant p53 proteins associated with the oncogenic phenotype (defined as mutant p53 gain-of-function), which have dominant-negative effects over wild-type p53, are degraded through a lysosomal-dependent pathway involving chaperone-mediated autophagy (CMA), a subtype of autophagy (defined and discussed in more detail later in this review) [16,17,18]. Thus, CMA activation can reduce the levels of accumulated mutant p53 and, thus, tumor-inducing phenotypes, such as those of drug resistance, cellular proliferation, survival, migration and cell invasion [17,18].

If DNA damage is too extensive or, for any reason, cannot be removed, chronic DDR signaling triggers cell death [19]. Although autophagy usually acts as a protective mechanism against DNA damage and other multiple stress stimuli, autophagy over a certain threshold may also trigger cell death [8]. The decision between an autophagy-mediated pro-death or pro-survival conduct also involves p53 [14].

The autophagic process can modulate specific DNA repair pathways, promoting DNA damage removal, and p53, as with other DDR mechanisms, can act as an intermediary of these autophagic effects on DNA repair. However, several recent reports also support a direct role of autophagy role in the control of DNA repair players, as will be presented below [2,3,4,5].

### 1.2. Autophagy: Mechanisms and Functions

Autophagy (Greek term that literally means self-eating) is a highly conserved cellular process that mediates degradation of intracellular compounds inside lysosomes. A diverse range of biomolecules (proteins, lipids, carbohydrates, and nucleic acids), organelles, and even intracellular pathogens can be degraded by this pathway [20,21]. Autophagy is stimulated for preserving cellular homeostasis in response to several stressful situations (nutrient deprivation, oxidative stress, and DNA damage, for instance) [22]. As a major protective mechanism, autophagy controls several cellular aspects, including: levels and quality of cellular compounds (preventing accumulation of abnormal proteins and organelles), energetic balance (recycling and providing important metabolic precursors), and cellular survival and death rates [23].

**Figure 1:**
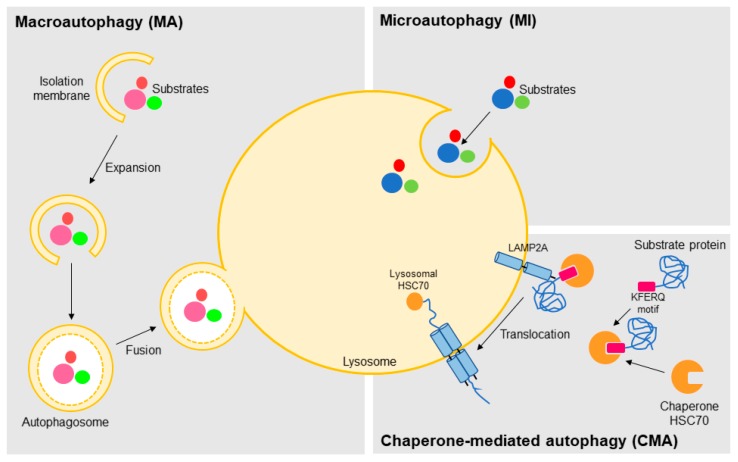
The different autophagy sub-types. Autophagy, which is a lysosomal-mediated degradation of intracellular compounds, is classified according to the way the cargo is carried to the lysosome lumen in macroautophagy (inside double-membrane vesicles termed autophagosomes), microautophagy (directly by local rearrangement of lysosomal membrane), and chaperone-mediated autophagy (by binding of the KFERQ motifs of the substrate proteins to the chaperone HSC70 (heat shock cognate protein of 70-kDa) and translocation across the lysosome mediated by the lysosome-associated membrane protein type 2A, LAMP2A).

Based on the pathway used for cargo delivery into the lysosome lumen, this degradative process can be classified in three distinct mechanisms: macroautophagy, microautophagy, and CMA (Figure 1) [24]. In macroautophagy, the best characterized autophagic process, the substrates targeted for degradation, which may include organelles, are sequestered in cytosol and carried to lysosome inside double-membrane vesicles termed autophagosomes [25]. Autophagosome biogenesis is highly inducible and it is carried out in three steps: initiation, nucleation, and expansion [26]. Although incompletely understood, many autophagy-related genes (ATG) have been identified and shown to be indispensable for this process [27]. After, the autophagosome fuses with a lysosome (autolysosome formation) leading to the degradation of the enclosed material and release of metabolic precursors, which in turn can be used either for macromolecules synthesis or as source of energy (Figure 1) [24,28].

In microautophagy, the substrates are directly taken by the lysosome through local deformation/rearrangement of its membrane. At present, little is known about the molecular mechanism and regulation of microautophagy [29,30]. Like macroautophagy, it is a nonselective process whereby a portion of the cytoplasm is delivered to the lysosome, thus being unable to mediate the selective degradation of individual proteins (Figure 1) [24,31]. On the other hand, CMA is a selective type of autophagy, so far only identified in mammalian cells, in which proteins are not delivered to lysosome inside vesicles, but rather identified and transported one-by-one by a cytosolic chaperone (predominantly the HSC70, heat shock cognate protein of 70-kDa) [31,32,33]. This selectivity is conferred by a specific sequence (KFERQ-like motifs) present in the target proteins. The recognition and translocation of this CMA substrate across the lysosome is mediated by LAMP2A (lysosome-associated membrane protein type 2A), which acts as a receptor for CMA substrates in the lysosome. Translocation of the substrate protein also requires the presence of an HSC70 form usually located in lysosome lumen (lys-HSC70) (Figure 1) [24,31,32,33]. Moreover, several reports have shown the existence of selective macroautophagy, where the protein p62 (also known as sequestosome 1, SQSTM1) acts as an adapter for the recognition of the autophagy substrates [34]. Similarly to the ubiquitin–proteasome system (UPS), ubiquitin is also the signal for degradation by macroautophagy [35].

Because of its central role in maintenance of cellular balance, autophagy is tightly regulated [36]. Thus, malfunction of autophagy has been related to several human disorders and diseases, such as neurodegenerative diseases, metabolic syndromes, premature aging, and cancer [33,37]. Moreover, accumulation of abnormal protein aggregates and dysfunctional mitochondria seems to be the basis of several neurodegenerative diseases, including Alzheimer’s disease, Parkinson’s disease, and tauopathies [38]. Therefore, the association between autophagy and neurodegeneration is not a surprise, given its essential function in the removal of aggregate-prone proteins and for preserving healthy mitochondria [39]. In mammals, autophagy was initially identified in liver cells, and since then many reports have supported its relevance in controlling liver metabolism and energetic balance [40]. Consequently, autophagy dysregulation is a common event in metabolic diseases and syndromes such as obesity, diabetes, and fatty liver [41]. Autophagy has also been implicated in aging, since the available methodologies for life span increase (some caloric restriction protocols, for instance) often stimulate this degradative process [42]. Moreover, premature aging is a common phenotype in organisms with genetic inhibition of autophagy [42].

The role of autophagy in cancer is complex, and depending on tissue type and tumor stage it can either inhibit or promote tumorigenesis and cancer progression [43]. In normal cells, autophagy acts as a barrier to limit tumor initiation. Thus, compromised autophagy leads to augmented cellular susceptibility to radical oxygen species (ROS) accumulation, DNA damage, and tumor transformation [44]. Supporting the role of autophagy as a tumor suppressor, the monoallelic deletion of the *Beclin 1* gene, the mammalian orthologue of yeast *Atg6* involved in autophagosome biogenesis, is detected in 40–75% of human sporadic cancers [45]. Heterozygous disruption of *Beclin 1* also increases the frequency of spontaneous malignancies in mice [45]. However, once tumors are established, autophagy activity supports the tumor survival in response to several challenges and stress situations, thus corroborating tumor maintenance and progression [44]. Moreover, autophagy provides metabolic precursors and helps tumor cells to sustain cellular growth even under nutrient deprivation conditions. Furthermore, in cancer cells, autophagy typically corroborates with the pro-tumor behavior increasing chemotherapy resistance.

As autophagy is frequently stimulated in response to many anticancer drugs in order to limit their effectiveness, it constitutes a potential target for cancer therapy. Therefore, the use of autophagy inhibitors combined with chemo- or radiotherapy has achieved encouraging results in several ongoing clinical trials [46,47]. However, there is still a long way to go for the effective use of autophagy inhibitors as an anticancer strategy, since autophagy can exert opposite roles in cell death control. Besides the positive role of autophagy in cellular survival processes, we cannot forget that the autophagic cell death is one of the three classical types of programmed cell death [48]. Therefore, the use of autophagy inducers has also been tested as a possible treatment for cancer. Exploring this autophagy paradox, it was recently demonstrated that simultaneous treatment with chloroquine (autophagy inhibitor), rapamycin (autophagy inducer), and an already established chemotherapy (such as vinorelbine) is a promising strategy for increasing cancer cell sensitivity to this drug [49].

### 1.3. DNA Repair Machinery

The integrity of DNA structure is continually subject to a range of aggressions that can cause damage and, consequently, perturb the maintenance of cell homeostasis. However, cells react to the presence of lesions in the DNA molecule activating biochemical pathways that regulate DDR according to the amount and the nature of DNA damage. These lesions can be endogenously induced, either by cellular metabolites or by products of cell respiration, such as radical oxygen species (ROS), or spontaneously, by hydrolysis of DNA. DNA damage may also be due to exogenous agents, such as ultraviolet light (UV) that causes bulky lesions, ionizing radiation causing DNA breaks, chemical reagents such as mustard gas, or chemotherapeutic drugs that induce adduct formation, breaks, and crosslinks, among others [50].

DDR involves an organized sequence of events activated by distinct and specific pathways, including DNA damage sensing and removal, signal transduction, chromatin rearrangement, cell cycle arrest and, finally, induction of cellular senescence or cell death. All these actions are important mechanisms which prevent genomic instability. DNA repair processes are activated in DDR and involve different biochemical pathways that can restore DNA integrity, aiming to preserve the cellular homeostasis [51].

Cells have many of these repair processes, which are classified basically in well-known mechanisms that either remove DNA damage or help the cells to tolerate them. Among these processes, homologous recombination (HR) and non-homologous end-joining (NHEJ), responsible for the repair of double strand breaks (DSBs) and interstrand cross links (ICLs), are the mechanisms where interaction with autophagy is better reported. However, autophagy is also related in different ways with three excision repair pathways: base excision repair (BER), nucleotide excision repair (NER), and mismatch repair (MMR).

DSBs can be a direct product of ionizing radiation, reactivity of ROS or several chemical agents with DNA. Alternatively, DSBs can occur as a consequence of collapse of arrested replication forks. These lesions may result in cell death and are handled by NHEJ and HR. HR generally occurs during late S to G2 phase of the cell cycle, as homologous DNA is provided as template for the precise recombination [52].

**Figure 2:**
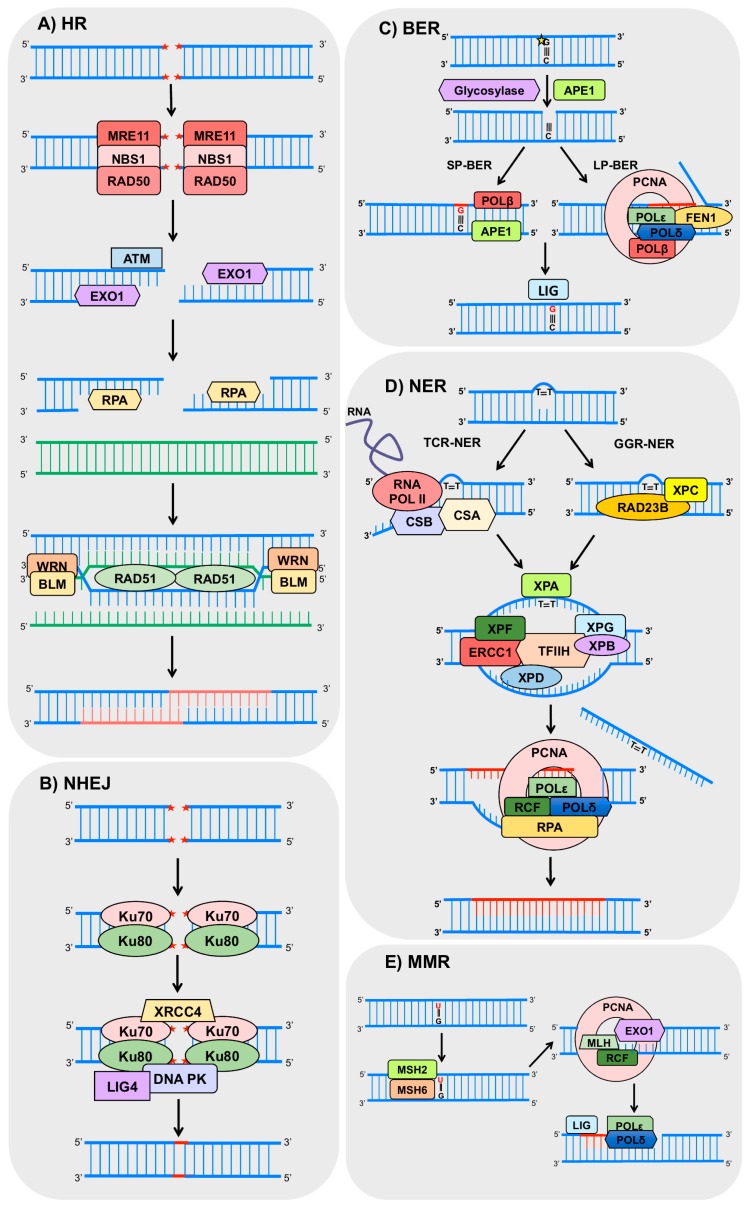
Schemes of the main DNA repair pathways reported to crosstalk with autophagy. The figures represent only the main steps for each pathway (indicated on the figure) and show only part of the proteins involved, focusing on those commented in the text to have a role on autophagy. HR (homologous recombination), NHEJ (non-homologous end joining), BER (base excision repair), NER (nucleotide excision repair), GGR (global genome repair), TCR (transcription-coupled repair), MMR (mismatch repair), MRE11 (double strand break repair, DSB, nuclease), RAD50 (human homolog of *S. cerevisiae* RAD50), NBS1 (Nijmegen breakage syndrome 1), ATM (ataxia telangiectasia mutated protein), EXO1 (exonuclease 1), RPA (replication factor A), WRN (Werner syndrome RecQ like helicase), BLM (Bloom syndrome REcQ like helicase), RAD51 (human homolog of *S. cerevisiae* RAD51), XRCC4 (X-ray cross complementing 4), DNA PK (DNA-dependent protein kinase), LIG4 (DNA ligase 4), APE1 (AP-endonuclease1), POLβ (DNA polymerase beta), POLε (DNA polymerase epsilon), POLδ (DNA polymerase delta), PCNA (proliferating cell nuclear antigen), FEN1 (flap endonuclease 1), LIG (DNA ligases), RNA POL II (RNA polymerase 2), CSA (Cockayne syndrome A), CSB (Cockayne syndrome B), XPC (xeroderma pigmentosum complementation group C), RAD23B: (human homolog B of *S. cerevisiae* RAD23), XPA (xeroderma pigmentosum complementation group A), XPF (xeroderma pigmentosum complementation group F), ERCC1 (excision repair cross-complementation group 1), TFIIH (transcription factor II H), XPB (xeroderma pigmentosum complementation group B), XPD (xeroderma pigmentosum complementation group D), XPG (xeroderma pigmentosum complementation group G), RCF (replication factor C), MSH2 (MutS homolog 2), MSH6 (MutS homolog 6), MLH (MutL homolog). The red stars represent DNA DSB lesions and the yellow star represents oxidized DNA base.

Basically, HR initiates by the recognition of the break by the proteins MRE11 (double strand break repair nuclease), RAD50 (human homolog of *S. cerevisiae* RAD50) and NBS1 (Nijmegen breakage syndrome 1), which together compose the MRN complex [53]. This complex recruits and activates the ATM (ataxia telangiectasia mutated) protein, responsible for the phosphorylation of downstream targets of a signaling pathway, that may arrest the cell cycle, allowing time for the correct execution of the DNA repair [54,55]. The MRN complex also recruits exonucleases (including EXO1, exonuclease 1), which promote resection at the DNA extremities to generate 3′-ssDNA overhangs [45]. The resulting ssDNA is stabilized by RPA (Replication Protein A) binding, which is later replaced by the recombinase RAD51 (human homolog of *S. cerevisiae* RAD51), forming a nucleoprotein filament [56]. This may also be stimulated by RAD52 (human homolog of *S. cerevisiae* RAD52) and BRCA2 (breast cancer 2) [57,58], and the ssDNA facilitates the invasion of the homologous DNA [52]. After this invasion, the resulting gaps are filled, and complex structures called Holliday junction are formed. Those structures are solved by helicases, such as WRN (Werner syndrome REcQ-like helicase) and BLM (Bloom syndrome REcQ-like helicase), resulting in crossover or non-crossover products [59,60] (Figure 2A).

NHEJ initiates with the binding of Ku70-Ku80 heterodimers to both ends of DSB, allowing the binding of the other protein complexes. Subsequently, DNA-PKcs (DNA-dependent protein kinases) and LIG4/XRCC4 (ligase 4/X-ray repair cross complementing 4) heterodimers are recruited to the damage site promoting the ligation of the two ends [61]. However, some rounds of resection and addition of nucleotides can occur in both ends of the break in order to promote microhomology between the ends as incompatible DNA ends are generated [61] (Figure 2B). Importantly, all these steps make NHEJ a very error-prone process [62].

BER is the main mechanism for the removal of small base alterations in the DNA structure such as oxidized bases, deamination, alkylation, and abasic (AP) site and single strand breaks [63,64]. Basically, the initial steps of BER involve base damage recognition and excision by a DNA glycosylase (such as OGG1, 8-oxoguanine DNA glycosylase) and cleavage of the phosphodiester chain at the AP site by an AP-endonuclease, such as APE1 (apurinic/apyrimidinic endonuclease 1). Then, the resulting gap is filled by a DNA polymerase (mostly POLβ, polymerase β), and finally, there is nick sealing by LIG3 and XRCC1 (DNA ligase 3 and X-ray cross complementing 1) [65,66,67]. Other enzymes, such as the PCNA (proliferating cell nuclear antigen), FEN1 (flap endonuclease 1), PARP1 (poly (ADP-ribose) polymerase 1), and LIG1 (ligase 1) also participate in BER [65,68] (Figure 2C).

The repair of bulky lesions that distort the DNA double helix structure, and block the progression of replicating and transcribing polymerases, is performed by NER [69]. This is the case of UV-induced DNA lesions such as cyclobutane pyrimidine dimers (CPDs) and 6-4 pyrimidine-pyrimidone (6-4PPs) [70]. Essential NER steps consist of: (1) damage recognition; (2) unwinding of the double helix; (3) incisions on both sides of the lesion and removal of the damaged strand; and (4) gap-filling synthesis followed by DNA ligation. NER can be divided in two sub-pathways depending on the location of the lesion: global genome repair (GGR) and transcription-coupled repair (TCR) [70,71]. GGR removes the lesions throughout the genome, when DNA damage is recognized by the complex XPC-RAD23B (xeroderma pigmentosum complementation group C—human homolog B of *S. cerevisiae* RAD23) [72]. This complex binds to the strand opposite to the damage and mediates the recruitment of the complex transcription factor II H (TFIIH) [71]. TCR occurs in active genes, when lesions stall the RNA polymerase II, and are recognized by the proteins CSA (Cockayne syndrome A) and CSB (Cockayne syndrome B). In both sub pathways the TFIIH complex is recruited and unwinds the DNA at the damage site, creating a bubble (approximately 30 bp) and allowing the recruitment of the XPA (xeroderma pigmentosum complementation group A) and the RPA complex. The endonucleases XPF/ERCC1 (xeroderma pigmentosum complementation group F/excision repair cross-complementation group 1) and XPG (xeroderma pigmentosum complementation group G) make the incision on both sides of the damaged strand and DNA polymerases δ and ε associated with RFC (replication factor C) and PCNA, respectively, are able to perform the gap-filling synthesis [73,74]. Finally, LIG1 or LIG3 performs the nick sealing [75] (Figure 2D). Interestingly, mutations in NER genes culminate in autosomal recessive human diseases such as xeroderma pigmentosum (XP), Cockayne syndrome (CS), and trichothiodystrophy (TTD).

MMR is another important mechanism responsible for the recognition and removal of base–base mismatches and insertion/deletions loops (IDL), which are products of errors in replication or homologous recombination, thus preventing base substitutions and repeat sequence instability [76,77,78]. In general, the pathway is initiated when the highly conserved proteins MSH2 and 6 (MutS homolog 2 and 6) recognize single base mismatches and 1 or 2 base IDLs. In cases of IDLs with two or more extra bases, MSH2 and MSH3 are responsible for the detection [79,80,81]. Following recognition, one of the MLH (MutL homolog) heterodimers binds to the mismatch, and PCNA is loaded onto DNA by replication factor C, activating MLH to incise the nascent strand and remove the error in an ATP-dependent manner [82,83]. Then, DNA polymerase δ [84,85] or ε [86] synthetizes the new strand followed by nick sealing [84] (Figure 2E).

Some chemical compounds react with DNA, generating covalent linkage between the opposite strands, the ICLs [87,88]. Those ICLs are extremely cytotoxic and the repair of these structures may involve several repair pathways, such as NER and HR, simultaneously. Specific proteins may also be involved in the repair of ICLs, and many are involved Faconi anemia (FA) syndrome. To date, more than 30 enzymes are thought to be involved in ICL repair, and more than 20 are linked to FA [89]. The ICL repair depends on the cell cycle phase and it is more critical when it occurs in S phase, depending on an even larger range of proteins [90]. The chromatin reorganization and the entire mechanism involved in ICL repair are still unclear.

## 2. Modulation of DNA Repair Pathways by Autophagy

While it is a cytoplasmic process, autophagy is able to control the DNA repair machinery by a diverse range of molecular mechanisms. Autophagy is known as critical for DSB repair, but more recent reports have also proved that key autophagy players are required for efficient removal of other DNA damage types. Consequently, autophagy plays important roles in distinct DNA repair pathways.

### 2.1. HR and NHEJ

**Figure 3:**
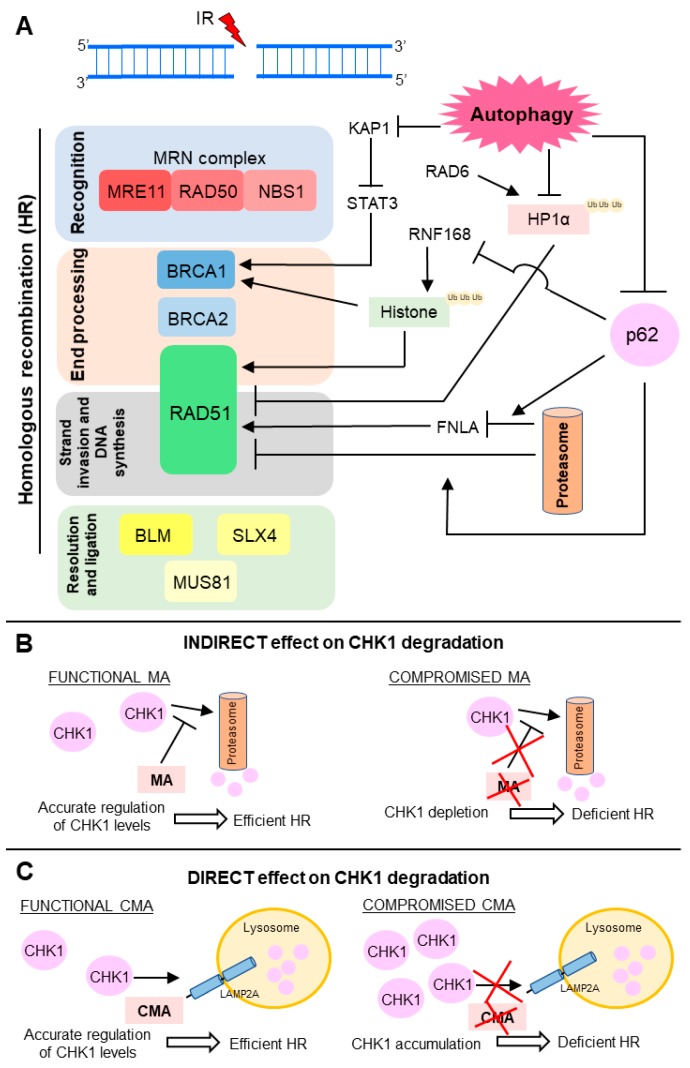
Scheme of the molecular mechanisms known for the autophagy-mediated regulation of double strand break (DSB) repair. (**A**) Main points of the HR modulation by autophagy. Autophagy degrades proteins KAP1 (KRAB (Kruppel-Associated Box Domain)-Associated Protein 1), heterochromatin protein 1 (HP1α) and p62) involved in negative modulation of BRCA1 (breast cancer 1) and RAD51, activating HR; (**B**,**C**) Autophagy controls CHK1 (checkpoint kinase 1) levels through (**B**) indirect and (**C**) direct mechanisms; (**B**) Macroautophagy (MA) down-modulates proteasome-mediated degradation of CHK1. Compromised MA depletes CHK1 levels and decreases HR activity; (**C**) Chaperone-mediated autophagy (CMA) mediates lysosomal degradation of CHK1. CMA deficiency leads to harmful CHK1 accumulation and HR blockage. IR: ionizing radiation.

Autophagy activity impacts on DSB repair by positive regulation of HR through distinct mechanisms (Figure 3) [4,5,7,91]. On the other hand, several studies suggest that autophagy does not exert any effect on NHEJ. However, the actual role of autophagy in NHEJ is still uncertain and under discussion. In this sense, by use of a conditional autophagy defective mouse model, Lin and colleagues showed that lack of autophagy led to down-modulation of proteins critical for HR, and also for NHEJ in hematopoietic cells [92]. Confirming these results, knockdown of *Beclin 1*, the UV radiation resistance-associated gene (*UVRAG*) or *ATG5* in human colon cancer cell lines enhanced ionizing radiation-induced DNA damage and cell death [93]. Down-regulation of these important autophagy players changes the percentage of irradiated cells with nuclear foci positive for TP53BP1 (tumor protein p53 binding protein 1), an NHEJ marker, but not Rad51, an HR marker [93]. These findings suggest that autophagy could also affect error-prone DSB repair, but so far, the molecular mechanism by which this autophagy-mediated regulation of NHEJ occurs has not yet been elucidated [92,93].

As a degradative process, autophagy can mediate direct degradation of molecules involved in DDR. Thus, autophagy protects bone marrow hematopoietic cells against radiation-induced genotoxic stress by directly targeting the KAP1 protein, a corepressor for the Kruppel-associated box-domain-containing zinc-finger proteins, for degradation [94,95]. Also, in response to DNA damage, ATM kinase phosphorylates KAP1, driving heterochromatin relaxation and repair [96,97]. Moreover, KAP1 is a repressor of STAT3 (signal transducer and activator of transcription 3) activation [94,95]. Hence, autophagy promotes repair of radiation-induced DNA damage through STAT3-mediated transcriptional up-regulation of BRCA1, a pleotropic DDR protein that functions in both checkpoint activation and DNA repair, mainly in DSB removal through HR (Figure 3A) [94,98].

Chen and collaborators revealed that autophagy degrades HP1α (heterochromatin protein 1) in response to X-ray irradiation [99]. Since the local disassembly of HP1α at DSB sites is crucial for RAD51 recruitment and effective HR, autophagy impairment leads to HP1α accumulation and disruption of this DNA repair pathway [99,100]. For HP1α, autophagy-mediated degradation it must be ubiquitinated at the K154 residue by RAD6, an E2 ubiquitin-conjugating enzyme induced in response to DNA damage and required for the repair of DSB and UV-induced DNA lesions [99,101]. Currently, selectivity mechanisms of macroautophagy have been proposed [102]. Several reports support that ubiquitination is often required for substrate recognition, thus determining macroautophagy selectivity in higher eukaryotes [103]. Therefore, in summary, upon irradiation, RAD6 interacts with HP1α and catalyzes its ubiquitination, which in turn promotes HP1α degradation by autophagy and subsequent changes in chromatin structure, allowing HR DSB repair (Figure 3A) [99].

The ubiquitin proteasome system (UPS) and autophagy are the two-major intracellular protein degradation processes and they are no longer viewed as independent or parallel mechanisms [28,104]. The ubiquitin code seems to be the link between these degradation pathways, assisting in choosing by which path the substrate must be degraded [103]. p62, also termed sequestosome 1 (SQSTM 1), mediates the crosstalk between autophagy and UPS through its non-covalent interaction with ubiquitin or polyubiquitin chains [7,35]. Besides being a classical receptor for macroautophagy, p62 also delivers ubiquitinated proteins to proteasomes and, it can be detected both in the cytoplasmic and nuclear compartments. p62 is a key mediator of the autophagy effect on DNA repair and it is able to control the ratio between HR and NHEJ. Upon irradiation, nuclear p62 co-localizes with DNA damage foci and increases proteasomal degradation of FLNA (filamin A) and RAD51 [7]. p62 can also regulate TP53BP1 foci, in a FLNA-dependent manner, but the mechanism is not yet understood. Thus, p62 accumulation, through autophagy blockage, suppresses RAD51-mediated HR and increase dependence on NHEJ. On the other hand, p62 down-modulation, as a result of autophagy stimulation, increases HR and reduces NHEJ efficiency (Figure 3A) [7].

Besides signaling to protein degradation, ubiquitination also plays a crucial role in DDR [105]. Induction of DSB leads to site-specific histone ubiquitination, which is necessary for further recruitment of downstream DNA repair factors, such as TP53BP1 and BRCA1 complex, which in turn contributes to NHEJ and HR choice [91,105]. Autophagy-deficient cells present decreased levels of DNA-damage-induced histone H2A ubiquitination. p62, which accumulates in cells with compromised autophagy, binds and inhibits E3 ligase RNF168 activity, resulting in reduced levels of histone H2A ubiquitination upon DNA damage, diminished DNA repair, and increased cellular sensitivity to radiation [6,91]. Thus, autophagy inhibition impaired BRCA1, RAP80, and RAD51 recruitment and, consequently, autophagy-deficient cells presented reduced efficiency of HR, but not NHEJ (Figure 3A) [6,91]. Since macroautophagy is required for DNA repair by HR, macroautophagy-deficient cells are more dependent on NHEJ. Therefore, inhibition of NHEJ following DNA damage can be a lethal synthetic strategy for killing autophagy-deficient cells [5,106].

Control of CHK1 (checkpoint kinase 1) levels is another important mechanism by which autophagy controls HR activity (Figure 3B,C) [106,107]. Indirectly, macroautophagy regulates the CHK1 levels through inhibition of its proteasome-mediated degradation (Figure 3B) [106]. Therefore, compromised macroautophagy leads to CHK1 depletion as a result of uncontrolled and intensified CHK1 degradation (Figure 3B). However, autophagy can also mediate direct degradation of this cell cycle checkpoint regulator and, consequently, control the HR-mediated DSB processing (Figure 3C) [107]. Thus, CMA, a selective autophagy subtype, tightly regulates CHK1 levels and prevents the MRE11-RAD50-NBS1 (MRN) complex hyperphosphorylation and destabilization [107]. Thus, in this case, CMA loss results in unnecessary and harmful CHK1 accumulation, causing HR deficiency (Figure 3C).

Well-known autophagy positive regulators have been implicated in DSB removal, but also through autophagy-independent mechanisms [108,109]. In this context, Beclin 1 protects cells from radiation-induced DNA damage, by an autophagy-independent manner, when relocated to the nucleus. In fact, during mouse development, and after exposure to ionizing radiation, Beclin 1 translocates from cytoplasm to nucleus. Knocking out of Beclin 1 reduced activity and levels of crucial repair proteins from both DSB damage repair mechanisms: HR and NHEJ. Furthermore, *Beclin 1* knockout attenuated the formation of the DNA-PK complex, with a slight effect on MRN, pivotal for initiation of NHEJ and HR, respectively. To mediate this effect on DNA repair machinery, Beclin 1 must be located in the nucleus. Complementation of these knockout cells with wild-type Beclin 1 recovered their cellular repair capacity. On the other hand, complementation with a mutated form of Beclin 1, lacking only nuclear localization domains, was unable to recover repair [108].

Initially identified for its capacity to partially complement the UV sensitivity in XP cells, UVRAG is a recognized tumor suppressor involved in autophagy induction by a positive regulation of Beclin 1-PI3KC3 complex, essential for the early stages of autophagosome formation [110,111]. It was demonstrated that UVRAG also promotes DNA DSB repair, but independently of its role in autophagy [109,112]. UVRAG deficiency leads to DNA DSB accumulation, since UVRAG is required for DNA-PK complex formation and the activity of this catalytic subunit [109]. Thus, through direct interaction with DNA-PK complex, UVRAG is essential for efficient NHEJ repair. Moreover, Beclin 1 and its cofactor UVRAG also control genomic stability, in an autophagy-independent manner, by maintaining centrosome stability [93,109,112].

### 2.2. BER

The relation between BER and autophagy is still poorly investigated and unclear. BER is the main pathway for the removal of base alterations including oxidation by ROS, and many studies indicate a relation between ROS generation/control and autophagy.

Despite the already-described protector role of autophagy in cells under stress conditions, Siggens and colleagues observed that nutrient deprivation and autophagy impaired BER in mammals’ cardiomyocytes by decreasing the protein levels of OGG1 glycosylase. However, there was no decrease in levels of other BER enzymes, such as PARP1 and APE1, caused by autophagy and starvation [113].

**Figure 4:**
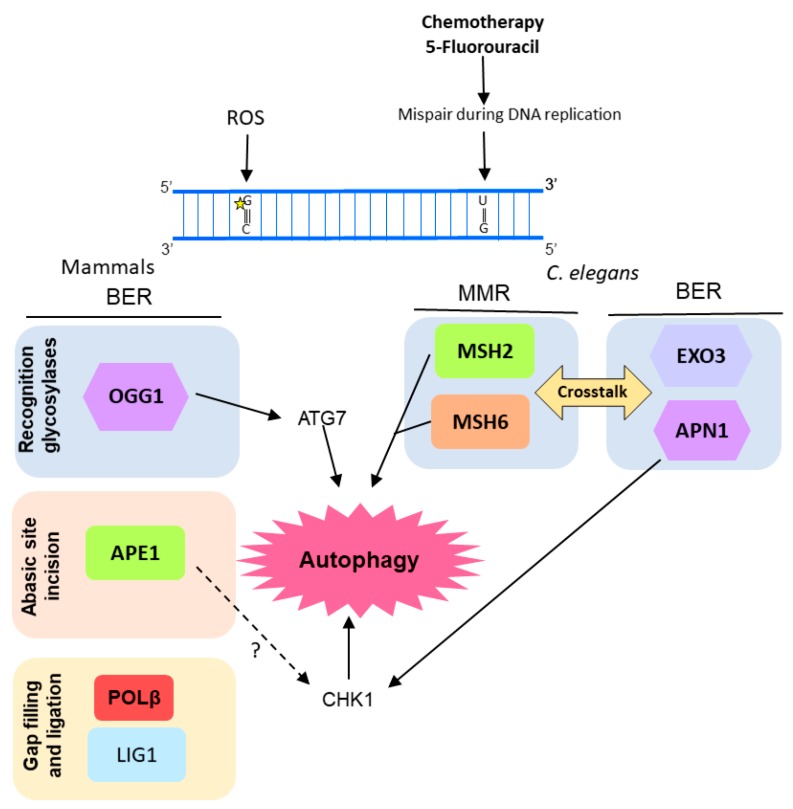
Diagram of the molecular mechanisms by which autophagy, BER, and MMR interacts in response to DNA lesions induced by reactive oxygen species (ROS) or chemotherapeutic agents. In human pulmonary cells, ROS-mediated DNA damage leads to an increase in OGG1 (8-oxoguanine glycosylase 1) expression and consequently autophagy (through interaction with ATG7, autophagy related 7). In *Caenorhabditis elegans* there is a crosstalk between BER and MMR, after induction of DNA damage by 5-fluorouracil (5-FU) that culminates in induction of autophagy and, eventually, cell death, probably mediated by the DNA damage response (DDR) protein CHK1. It is still an open question whether human APE1 also regulates CHK1 and it is represented by a question mark (?) in the figure.

OGG1 was also associated with autophagy in another study using pulmonary cells. Ye et al. [114] reported a correlation among OGG1 expression, autophagy, and inflammatory response in mice cells under hyperoxia condition. They observed that lung cells of mice exposed to 95% O_2_ showed increased ROS-mediated DNA damage, inflammatory markers, and OGG1 protein accumulation. On the other hand, the OGG1 knockout mice presented an increased inflammatory response under hyperoxia when compared with wild type mice, as well as a reduction in autophagy-related proteins such as ATG7 and LC3B (microtubule associated protein 1 light chain 3 beta). Similarly, murine cells transfected with OGG1 siRNA and exposed to hyperoxia for 48 h showed down-regulation of ATG7 expression. Moreover, under hyperoxia conditions, OGG1 was shown to increase interaction with ATG7 protein and to control the ATG7 expression through direct interaction with its gene promoter (Figure 4).

Activation of autophagy was observed after induction of DNA damage by 5-fluorouracil (5-FU, an anti-cancer drug) in *Caenorhabditis elegans* embryos, as a consequence of the crosstalk between BER and MMR [103]. MMR proteins MSH2 and MSH6 act as sensors of DNA damage induced by 5-FU. Subsequently, *C. elegans* BERs AP endonuclease, APN-1, and EXO3, act in the same pathway inducing 5-FU toxicity, activating DDR by the phosphorylation of CHK-1, and leading to cell death by autophagy (Figure 4). Moreover, the human APE1 was also shown to play important role in the autophagy induction promoted by 5-FU in osteosarcoma cells (U2OS) [115].

### 2.3. NER

**Figure 5:**
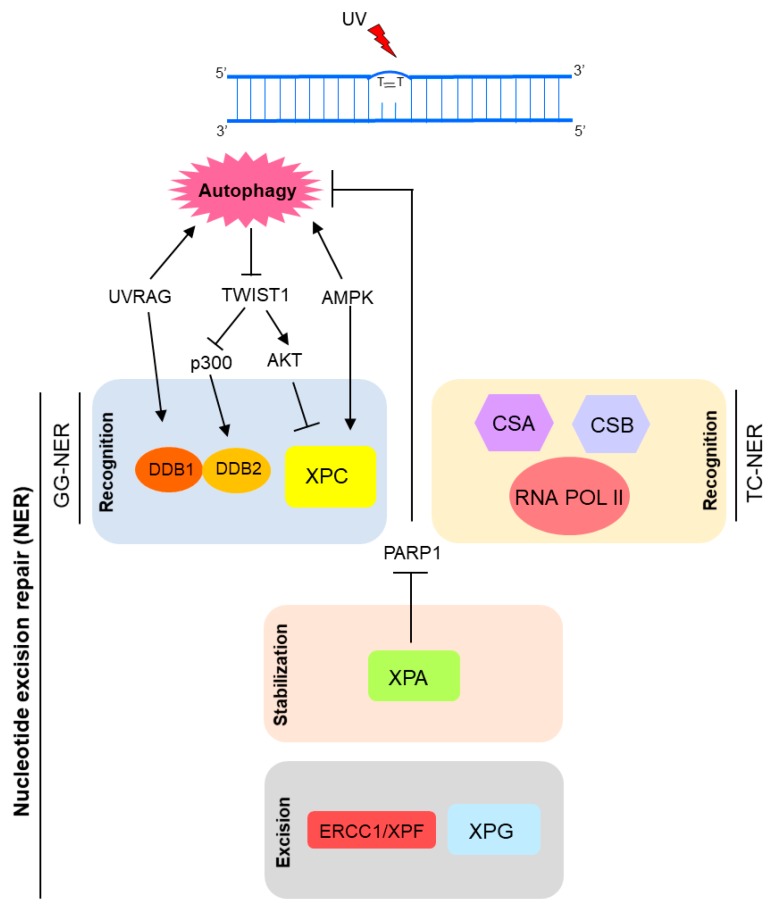
Model of NER regulation by autophagy. Autophagy controls crucial molecular players of the recognition step of the global genome (GG) NER, including damaged-DNA binding protein (DDB)1, DDB2, and XPC. Moreover, XPA (also essential for transcription-coupled (TC) NER) can induce autophagy by inhibition of PARP1 (poly(ADP-ribose) polymerase 1).

Recently, autophagy has also been implicated in NER modulation [2,3,116]. Autophagy seems to positively regulate UV-induced photolesions (CPD and 6-4PP) repair by two distinct mechanisms: upregulating XPC transcriptional expression and promoting DNA damage recognition by damaged-DNA binding protein 2 (DDB2), encoded by the *XPE* gene [3]. Thus, autophagy controls the global genome NER (GG-NER) subpathway initiation (Figure 4). Autophagy deficiency through loss of the key autophagy *Atg5* gene led to significant reduction of DNA damage repair and XPC protein levels [3]. Similarly, deletion of AMP active protein kinase (AMPK), a well-known autophagy inducer, down-modulated XPC expression and UVB-induced DNA repair [117] (Figure 5). In agreement with this new autophagy protagonism on UV-induced DNA damage repair, pharmacological modulators of this degradative cellular process interfered with the UVB-induced tumorigenesis [3]. Therefore, inhibition of autophagy with Spautin-1 induced new tumor formation and growth of established tumors in SKH-1 mice exposed to UVB. Interestingly, rapamycin, a classical autophagy inducer, showed the reverse effect on skin carcinogenesis [3].

The oncogenic transcription factor Twist1 is central for this autophagy-dependent NER activity. Autophagy deficiency induced Twist1 accumulation, which in turn led to XPC mRNA expression suppression by activation of E2F4-RBL2 (E2F transcription factor 4-Retinoblastoma-like protein 2) transcriptional repressor complex through AKT1 (AKT serine/threonine kinase 1) signaling [3,104]. This effect on XPC expression levels is relevant for both 6-4PP and CPD autophagy-mediated repair. Moreover, lack of autophagy also interferes with CPD repair by impairment of DDB2 recruitment to DNA lesion sites, in a Twist1-dependent manner. In an autophagy-deficient condition, Twist1 is stabilized by p62, leading to p300 inhibition, essential for DNA damage recognition by DDB2 (Figure 5) [3,116,118].

Besides the previous mentioned role of UVRAG in DNA DSB repair promotion, in an autophagy-independent manner, recently it was shown that UVRAG is important for NER activity. Since its isolation in 1997, the UVRAG association to UV-induced DNA damage was an open question until recently. Yang and colleagues have just demonstrated that UVRAG is recruited to the damage foci after UV exposure, and specifically interacts with the UV-induced photolesion sensor DDB1 in vivo [119], which together with DDB2, checks the whole genome for damage independently of transcriptional status. Thus, UVRAG promotes assembly and activity of the DDB2-DDB1-Cul4A-Roc1 (CRL4^DDB2^) ubiquitin ligase complex, which in turn performs histone ubiquitination and recruitment of chromatin remodelers and XPC protein to UV-induced lesion sites [119]. Therefore, UVRAG inactivation disrupts the UV-DDB-Cul4-Roc1-XPC axis, leading to impairment of GGR NER, genomic instability and tumorigenesis increase (Figure 5) [119].

This crucial role of UVRAG on UV-induced lesions repair must be further investigated, given its potential influence in melanoma predisposition and progression. It seems clear that there is an UVRAG association with UV-induced mutagenesis, since increased UV-signature mutations in melanoma correlate with reduced expression levels of this autophagy player [2,119]. Moreover, UVRAG patrols centrosome stability and DSB repair activity through direct binding to DNA-PK in NHEJ, standard mechanisms involved in cancer progression prevention [109,112]. As a bona fide genome guardian, UVRAG is an attractive target for cancer therapy [109,112].

Moreover, XPA, a key protein in NER pathway, has been implicated in autophagy upregulation in a DNA repair-independent manner [120,121]. Changes in autophagic levels promoted by XPA seem to be associated with chemotherapy resistance in cancer cells and may be related to the severe neurodegeneration phenotype observed in some DNA repair disorders [120]. Increased expression of XPA detected in some tumor cells is correlated with resistance to anticancer drugs, such as cisplatin. Thus, XPA knockdown sensitizes melanoma cells to cisplatin by impairment of autophagy through activation of PARP-1 [120]. Therefore, resistance of melanoma cells to cisplatin could be modulated by targeting the XPA-PARP1-autophagy pathway (Figure 5). On the other hand, XP-A (NER-related syndrome caused by genetic alteration in the *XPA* gene) patients present defective or null XPA expression and activity. Cells from these patients display mitochondrial dysfunctions related to SIRT1 (sirtuin 1) inhibition, a common mechanism for DNA repair disorders (XP-A and CS) with neurodegeneration. Hyperactivation of PARP-1 appears to be the cause of SIRT1 and mitophagy attenuation in XPA deficient cells [121,122].

### 2.4. MMR

So far, the implication of autophagy directly in DNA MMR has not been yet reported. Instead, a functional MMR system is required for autophagy activation in response to some chemotherapy drugs, particularly the nucleoside analogs 6-thioguanine (6-TG) and 5-FU [123,124,125]. Isogenic pairs of MMR-deficient and -proficient cancer cells were used in these studies. Activation of autophagy by 6-TG and 5-FU was detected only in cells positive for MLH1 and MSH2, molecules responsible for DNA mismatch recognition and ensuing repair. Zheng and Kinsella showed that this process is dependent on p53 activation, since its down-modulation blocked 6-TG-induced autophagy in MMR-positive cells [124,125]. More recently, Bcl-2 nineteen-kilodalton interacting protein 3 (BNIP3), a proapoptotic member of BH3-only subfamily involved in autophagy induction, also proved to be essential for autophagy induction following MMR processing [123,126]. Most probably, this MMR-initiated autophagy prevents chemotherapy-induced apoptosis, acting as a pro-survival mechanism in tumor cells [123,124,125].

### 2.5. Other DNA Repair-Related Pathways

Autophagy is also related to other molecular mechanisms important for genomic stability control, such as the FA pathway. Recently, Sumpter and colleagues demonstrated that some FA proteins are essential for two forms of selective macroautophagy: virophagy (selective for virus) and mitophagy (selective for mitochondria) [127]. The FANCC (Fanconi anemia complementation group C) protein is required for virophagy, but not for starvation-induced autophagy (a non-selective macroautophagy). Therefore, FANCC deficiency increases susceptibility to some neurotropic viruses [127]. Moreover, FANCC is also essential for clearance of damaged mitochondria by mitophagy, through its interaction with Parkin, well-known mitophagy mediator [127,128]. Besides FANCC, other proteins from FA pathway were reported to be essential for mitophagy, including FANCA, FANCF, FANCD2, FANCL, BRCA1 and BRCA2 [127]. This FA-mediated effect on autophagy could contribute the FA clinical phenotypes, including high cancer incidence in patients with mutations in *FA* genes [127].

## 3. Concluding Remarks

There is a clear crosstalk between autophagy and DNA repair. Therefore, malfunction of one them leads to deregulation of the other, and vice versa. This leads to important consequences for the cells and organisms. Problems in autophagy in patients with DNA repair defects may be linked to clinical phenotypes such as premature aging, developmental problems, and neurodegeneration. In this context, the NER-deficient and XPA-deficient cells present defective mitophagy and accumulation of dysfunctional mitochondria, which in turn may be associated to the neurodegenerative phenotype observed in XPA-deficient patients [121,122]. Thus, better understanding this crosstalk may help to comprehend the origin of these phenotypes (including premature aging) in patients, as well as how normal aging occurs. Hopefully this knowledge will reveal ways to improve the quality of life of DNA repair deficient patients and their families, and of the general population. On the other hand, autophagy down-modulation leads to failure of DNA repair, especially HR and NER [3,7,83,97]. Regarding DSB repair, lack of autophagy induces impairment of HR and super-dependence on NHEJ [7,97] creating a synthetic lethal situation in cell autophagy-deficient exposure to DSB inducers with cotreatment with an NHEJ inhibitor [97]. Moreover, the reports regarding the autophagy role on DNA repair suggest that the well-known chemotherapy resistance effect mediated by autophagy in cancer cells might be also a result of its positive effect on DNA repair of chemotherapy-induced DNA lesions. Thus, targeting autophagy by chemical inhibition, combined with already available chemotherapy genotoxic protocols, emerges as good strategy for potentiating anticancer treatment.

## Abbreviations

AMPK	AMP-activated protein kinase
AP	Abasic
APE1	Apurinic/apyrimidinic endonuclease 1
ATG	Autophagy-related
ATM	Ataxia telangiectasia mutated
BER	Base excision repair
BLM	Bloom syndrome REcQ like helicase
BNIP3	Bcl-2 nineteen-kilodaltoni protein 3
BRCA1 and 2	Breast cancer 1 and 2, respectively
CHK1	Checkpoint kinase 1
CMA	Chaperone-mediated autophagy
CPD	Cyclobutane pyrimidine dimers
CS	Cockayne syndrome
CSA and CSB	Cockayne syndrome A and B, respectively
DDB1 and 2	Damage-DNA binding protein 1 and 2, respectively
DDR	DNA damage Response
DNA-PK	DNA-dependent protein kinase
DRAM	Damage-regulated autophagy modulator
DSB	DNA double-strand break
ERCC1	Excision repair cross-complementation group 1
E2F4-RBL2	E2F transcription factor 4-Retinoblastoma-like protein 2
EXO 1	Exonuclease 1
FA	Fanconi anemia
FEN1	Flap endonuclease 1
FLA	Filamin A
GGR	Global genome repair
HP1α	Heterochromatin protein 1 Alfa
HR	Homologous recombination
HSC70	heat shock cognate protein of 70-kDa
ICL	Interstrand DNA crosslink
IDL	Insertion/deletions loops
KAP1	Corepressor for the Kruppel-associated box-domain-containing zinc-finger proteins, for degradation
IDL	Insertion/deletion loops
KAP1	KRAB (Kruppel-Associated Box Domain)-Associated Protein 1
LAMP2A	Lysosome-associated membrane protein type 2A
LC3B	Microtubule associated protein 1 light chain 3 beta
LIG1	DNA ligase 1
LIG3	DNA ligase 3
LIG4	DNA ligase 4
MLH	MutL homolog
MMR	Mismatch repair
MRE11	Double strand break repair nuclease
MSH2, 3 and 6	MutS homolog 2, 3 and 6, respectively
NBS1	Nijmegen breakage syndrome 1
NER	Nucleotide excision repair
NHEJ	Non-homologous end joining
OGG1	8-oxoguanine glycosylase 1
PARP 1	Poly (ADP-Ribose) polymerase 1
PCNA	Proliferating cell nuclear antigen
POLβ, ε and δ	DNA polymerase beta, epsilon and delta, respectively
RAD23B	Human homolog B of *S. cerevisiae* RAD23
RAD50, 51 and 52	Human homolog of *S. cerevisiae* RAD50, RAD51 and RAD52, respectively
RFC	Replication factor C
RNA POL II	RNA polymerase II
ROS	Reactive oxygen species
RPA	Replication Protein A
SIRT1	Sirtuin 1
SQSTM1	Sequestosome 1
SSB	DNA single-strand breaks
STAT3	Signal transducer and activator of transcription 3
TCR	Transcription coupled repair
TP53BP1	Tumor protein p53 binding protein 1
TTD	Trichothiodystrophy
TFIIH	Transcription factor IIH
UPS	Ubiquitin proteasome system
UV	Ultraviolet
UVRAG	UV radiation resistance-associated gene
XPA, B, C, D, F and G	Xeroderma Pigmentosum complementation group A, B, C, D, F and G, respectively
XRCC1 and 4	X-ray repair cross complementing 1 and 4, respectively
WRN	Werner syndrome RecQ like helicase
5-FU	5-fluoracil
6-4PP	6-4 pyrimidine pyrimidone
6-TG	6-thioguanine
